# Nanoparticles and Other Nanostructures and the Control of Pathogens: From Bench to Vaccines

**DOI:** 10.3390/ijms24109063

**Published:** 2023-05-21

**Authors:** Ariane Boudier, Alain Le Faou

**Affiliations:** 1 Faculty of Pharmacy, Université de Lorraine, F-54000 Nancy, France; ariane.boudier@univ-lorraine.fr; 2 Faculty of Medicine, Maieutic and Health Sciences, University of Lorraine, Pole Brabois Santé, F-54000 Nancy, France

Parasites and microorganisms (protozoa, bacteria, and viruses) are still a concern despite progress in hygiene and anti-infectious therapy. Humans are continuously exposed to these pathogens regardless of whether they are specifically of human or animal origin or from the environment. These infections, in addition to the ones observed in hospital settings, occur in everyday life as well. Thus, they are becoming more and more of a concern for a multitude of reasons.

Most of the recent new human infections are of animal origin (e.g., Mpox) and the occurrence of infections caused by similar sources is a concern (e.g., avian flu.) Pathogens (and their vectors), which are present in specific areas such as tropical zones, continue to extend their area of impact because of the ongoing climatic crisis (e.g., chikungunya and *Aedes albopictus* [1]). The widespread overuse of antimicrobial drugs and the aggressive treatment of immunocompromised hosts are responsible for the emergence of resistant microorganisms which are able to spread outside of hospital settings.

The human way of life permits easier pathogen circulation between individuals and different countries, thus provoking not only local epidemics but also dramatic pandemics (COVID-19). The pathogens responsible may become endemic in the colonized population. If the resistance to antimicrobial agents becomes a serious health problem for the treatment of diseases caused by bacteria and fungi, the absence of treatment or the limited number of new available molecules render the control of viral infections difficult. Viruses responsible for mild symptoms or that do not present symptoms in healthy individuals become life threatening in immunocompromised patients (the cytomegalovirus and the JC virus), and their occurrence is difficult to prevent and treat. Viruses are even able to develop resistance, as is the case for HIV, for which new drugs are, fortunately, still made available for treatment and prevention [2].

The introduction of new pathogens or the spread of previously seldom described infections (e.g., viral zoonosis, Table 1) occurs continuously and some examples are briefly described below:

− *Candida auris*: This emerging yeast, responsible for life-threatening infections, is in most cases resistant to anti-fungal treatment. It is more and more frequently described in hospitals [3].
− *Enterococcus faecium*: A species of commensal human bacteria that is found commonly in hospitals where it presents established or rapidly acquired resistance to vancomycin, the antibiotic usually utilized to fight the infection it causes. The antibiotics used as a last resort are quinupristin–dalfopristin and linezolid, which are responsible for adverse reactions, and two more antibiotics, daptomycin and tigecycline, for which interest has yet to be ascertained in such indications [4].
− Methicillin-resistant *Staphylococcus aureus* (MRSA): These strains, which are resistant to all β-lactamin antibiotics, are commonly found not only in hospital settings but also in animal settings, mainly on pig-raising farms. These bacteria may also acquire resistance to tetracycline, aminoglycoside, and trimethoprim. Moreover, they may harbor resistance-gene-containing plasmids from other species, such as vancomycin resistance from Enterococcus. The exchange of strains between humans and animals is common as well as human-to-human exchange, and this exchange demonstrates that these resistant strains are widespread. Alternative treatments are linezolid and pleuromutilins, which both inhibit protein production [5].
− *Monkeypox virus*: This zoonotic poxvirus, initially limited to tropical forests of central and west Africa, was, until recently, incidentally described in the local population. This virus is responsible for infections that may be deadly, particularly for patients covered with epithelial pustules. It has recently been found to be responsible for a worldwide human pandemic, and its proliferation has been limited by an antiviral drug (tecovirimat), a vaccine (Jynneos vaccine: the smallpox and monkeypox viruses, live and non-replicating), and hygiene measures. The animal reservoir of the monkeypox virus is not known but may be an animal from a rodent population.
− Severe-acute-respiratory-syndrome-related coronarius 2 (SARS CoV-2): This recent and still circulating infection (Covid-19) is responsible for a pandemic that affected millions of individuals all over the world [6]. Infected individuals develop an acute respiratory infection which may require hospitalization in intensive care units with life-threatening evolution. Although it is not known where the virus originated, its origin is almost certainly a bat population; however, how the passage from animals to humans is not yet understood [7]. Antiviral treatments (Paxlovid (nirmatrelvir/ritonavir) and molnupiravir) and vaccination reduce the risk of severe outcomes.

As a consequence, antimicrobial treatments, either for cure or prevention, are becoming less and less effective. Only a limited number of new drugs have become available, against which resistance may occur rapidly. To control viral infections, with AIDS and viral hepatitis B and C disregarded, only a limited number of antivirals are available for either chronic (e.g., herpes virus infections) or acute (e.g., influenza) infections, and in the case of acute episodes, they have to be administered as soon as possible or given for prevention. For protection against viral diseases, vaccinations have brought great success (e.g., smallpox eradication). The development of vaccines against bacteria and parasites continues and some of the results produced so far are promising. Although vaccines against tetanus or diphtheria have been administered for many years and have almost led to the disappearance of these two diseases, some severe diseases are still widespread and their eradication remains to be tackled. However, considering serious and very common infections, in tropical countries, vaccines with some limitations regarding their efficacy are nonetheless used. The only actual efficient prophylactic weapon against malaria due to *Plasmodium falciparum* is RTSS, S Mosquirix^®^ (a repeated T epitope (RTS) derived from PfCSP combined with the S antigen derived from the hepatitis B surface antigen (HBSAg) and the AS01-liposome-based proprietary adjuvant) is administered to infants, despite its limited efficacy (30% fewer hospitalizations). Another vaccine, the recently proposed R21/Matrix-M™ (R21/MM), using a different adjuvant (Matrix-M, proprietary adjuvant) may have better efficacy [8] and has been recently approved in Ghana and Nigeria [9]. The administration of the tetravalent dengue vaccine, Dengvaxia^®^ (four chimeric vaccinal recombinant strains of *yellow fever virus* each expressing antigens of one of the four types of Dengue virus), is strictly limited to children aged from 9 to 16 years with laboratory-confirmed previous dengue virus infection and living in an endemic area [10].

Thus, new efficient tools have become necessary to overcome the increasing prevalence of microbial resistance and to prevent (or limit) the spread of infectious diseases. For this purpose, nano-objects have proved to be very valuable due not only to their size, adapted to cellular or intracellular environments, but also their almost infinite possibility of composition, which permits them to achieve effective antimicrobial action. Moreover, specific targeting or enhancement of organism defenses could be obtained by immunostimulation or vaccination.

The relationships between nano-objects and microorganisms are the subject of numerous scientific studies whose goals are, for the most part, to improve or provide more advanced tools for the fight against pathogens. NPs, for example, are already used for this purpose. They were first used as liposomes to limit the toxicity of drugs (e.g., amphotericin B) but have since been made available for several applications. Due to its antimicrobial properties, NP-Ag is included in wound dressings [11]. NPs have entered the field of diagnosis for imagery [12] and in laboratory settings (e.g., rapid tests using immunochromatography with Au-NP). In recent years, several NP-containing vaccines have been designed to protect individuals from COVID-19, with their method of action being bringing mRNA into cells, which encodes for the “spike protein” (Comirnaty^®^ and Spikevax^®^) and the recombinant “spike protein” being included in a nano-structure (Nuvaxovid^®^, VidPrevtyn Beta^®^, and Bimervax^®^). Owing to their size, viruses may be considered as NPs even if they differ in belonging to the living world. COVID-19 vaccines use inactivated SARS-coronavirus-2 particles (Valneva^®^) or recombinant adenovirus whose DNA encodes the “spike protein” (Vaxzevria^®^ and Jcovden^®^).

Bacteriophages have even been proposed and have been used for many years in the treatment of local chronic bacterial infections for which antibiotics are, in many cases, inefficient (vesical or bone infections).

Thus, nano-objects have already been proven useful as new tools to fight infections. Some materials, such as colloidal silver, are already administered, mainly for external use, and others, such as more complex materials containing metals (or their oxides) with known antimicrobial properties (gold, copper, and zinc), are regularly proposed for future in vivo use [12]. The functionalization of nano-objects, specifically addressing microbial structures (just as bacteriophages do) or cell surface proteins or using magnetic properties to direct them to the site of action under a strong magnetic field, are devised for medical use (diagnosis, treatment, and theragnostics) [13]. They offer new ways to control pathogens not only in medicine but also in food processing [14] as well as in the environment [15]. For example, the prevention of bacterial growth has gained great importance in fresh processed food to preserve taste and protect customers from infections while the products remain on refrigerated shelves for days. Nanoparticles are already added to food wrapping materials for this purpose and examples include: Ag-NP, TiO_2_-NP, and ZnO-NP. The utilization of some is pending due to possible carcinogenic activity (TiO_2_-NP).

The field of the relationship between NPs and microorganisms is very wide and is reflected in the seven articles included in the present Special Issue. This topic area is incredibly vast, with it covering the design of NPs with an antimicrobial component, the interactions between NPs and microorganisms, the antimicrobial properties of NPs, and their utilization in protection against infections.

1. The review by T. Nagabuko [16] deals with interest in the properties of phage-tail-like structures that are encoded by the bacterial genome. These structures are akin to tailed phages. As a matter of fact, *TequarovirusT4* (the subspecies of *Tevenvirinae*), for example, has its genome packed into the phage head. The tail consists of a tube inside a contractile sheath, a base plate to which six arms are attached, and a terminal spike. After attachment of the phage to the bacterium surface, the extremity of the tube reaches and penetrates the cytoplasmic membrane, thanks to the sheath contracting, and the entire viral genome is ejected (“injection”) into the cytoplasm through the tube, initiating the infectious cycle. The three types of phage-tail-like structures function according to the same mode as the phage tails but are protein-only structures.
  - The eCIS (external cellular injection system) recognizes the bacteria and insect cells [17] and delivers a toxin after piercing the cellular membrane.
  - The T6SS (type 6 excretion system) has antimicrobial activity (bacteria and fungi). In *Pseudomonas aeruginosa*, a toxin (Ts2) is introduced into the cytoplasm [18].
  - Tailocins are considered bacteriocins. They act by disrupting the activity of the proton motive force. The tube allows for the entry of protons into the cell, thus bypassing ATP synthase [19].
  All three structures, which may co-exist in the same bacterium, play an important role in the interaction of a given bacterial species with other challenging strains, sometimes fungi (eCIS and T6SS) and insects (eCIS). These structures act in the same way as the first step of phage infection and are thus models of nanomachines [19].
2. The article by J.C. Pieretti et al. [20] deals with the physicochemical study of NPs consisting of a magnetite core covered with two successive layers of Ag-NP first and then chitosan for its stabilizing properties. The structure and interaction of the NPs during the three synthesis steps were investigated. The interactions of these three particles with albumins were also studied. The presence of Ag-NP gives this particle antimicrobial activity. This is of importance as albumin is the major protein found in the blood. These interactions are far less limited after adding a chitosan layer which ensures a lesser probability of modification of the albumin structure and the free NPs are thus more available for putative therapeutic use.
3. The study by B. Kiss et al. [21] showed the interaction of a single T7 phage (*TeseptimavirusT7*, subfamily *Studiervirinae*) particle with a bacterium. The first contact between the two entities is reversible until the tail fiber recognizes its receptor; then, the phage is fixed, and the infectious process begins, ending with the lysis of the bacterial cell and the liberation of virus progeny. This process has been studied in full. The first step is the establishment of the location of their receptor by the six phage fibers which occurs through a trial–failure process and random movement of the particle at the cell surface until the receptor is found. Thus, the displacement of the T7 phage provides a model of a nanomachine.
4. The study by E. Tomaszewska et al. [22] presents a model of antiviral therapy using metallic NPs (Ag- and Au-NP). HSV1 and HSV2 (*Human alphaherpesvirus 1* and *2*; genus *Simplexvirus*, subfamily *Alphaherpesvirinae*) are both responsible for persistent infections as they establish a latency state inside ganglia neurons from which recurrent infection occurs more or less frequently. Immunocompromized hosts are at risk of disseminated infections with viral spread in the organism which may be associated with life-threatening encephalitis. One way to control the infection is to prevent the recognition of its cell surface receptor by the virus. While several therapeutic molecules have been proposed, the authors present an original approach using metallic NPs coated with tannic acid. Au-NP has been shown to demonstrate the same antiviral activity as Ag-NP for 5 nm-sized NPs but for 30 nm NPs, the activity of Ag-NP remains similar. Additionally, the activity of Au-NP is reduced. The addition of a sulfonate ligand to the NP reduced the antiviral activity of the NP as compared to tannic acid. However, the role of the pure metallic NPs is not shown. Would tannic acid alone be virucidal? In the study, there is no demonstration of a synergistic effect. For these studies, pretreatment was applied in vitro (1 h at +4 °C). Ag-NP inhibits HSV2 after cell pretreatment for 24 h [23]. Tannic-acid-coated Ag-NP embedded in a hydrogel has been shown to induce inhibition of HSV replication using a murine model [24]. Thus, the addition of tannic acid to noble metal NPs may be an interesting antiviral tool.
5. T.A. Adekiya et al. [25] evaluated lipidic NPs loaded with praziquantel for the treatment of *Schistosoma mansoni* colonization of the murine gut. *S. mansoni* is responsible for the most widespread intestinal schistosomiasis and is very common in the tropical countries of Africa and America. The infection is chronic and lasts for years. Praziquantel is administered for treatment and prophylaxis. This study proposes stabilization of the drug by loading it inside of solid lipid NPs. In vitro, the drug is released progressively over 24 h, and its construction is stable for weeks. This complex has been studied in an infected murine model and was proven to be devoid of toxicity and ensured antiparasitic activity 2 and 4 weeks after animal infection following oral administration while limiting drug toxicity. Such a long stability period for the complex and the progressive release of praziquantel would aid in the fight against this parasite.
6. The study by F. Mancini et al. [26] proposes a vaccine substrate to protect against *Shigella sonnei* and *Shigella flexneri* which are causative agents of severe diarrhea, mainly in developing countries, and may present antibiotic resistance. In the study, it is stated, “O-antigen-based vaccines may not be the best solution if one refers to the former *Salmonella typhi* vaccine, which is almost devoid of a protective effect.” Thus, their proposal of using generalized modules for membrane antigens (GMMAs) might be of value. They consist of outer membrane vesicles containing both the O-antigen and proteins from non-toxin-producing strains. The latter may contribute to boosting immunity against O-antigens by stimulating T-cell helpers while, at the same time, eliciting specific antibodies. Trials have been conducted by immunizing mice with two intramuscular shots of GGMA from *S. sonnei* and three serovars of *S. flexneri* at 4-week intervals. Mouse mutants (TLR4^mut^/(LPS) and TLR2-/- C3H mice) have provided evidence of the role of TLR4 and TLR2 agonists in T-cell-deficient mice (Crl:CD1-Foxn1^nu^) and the role of proteins in GMMA immunogenicity. The results are in favor of the vaccinal efficacy of GMMAs, which have been shown to be devoid of toxicity in phase I/II trials in humans, and thus serve as a basis for a future vaccine against Shigella infections.
7. C. Monge et al. [27] used a model of a sublingual vaccine and investigated the reaction of mice following the addition of an adjuvant to evaluate the feasibility of such a procedure. They compared oral liquid administration and a mucosa-adherent chitosan patch containing NPs. These NPs were composed of polylactic acid in which adjuvants (telratolimod (3M-052, the agonist of TLR 7/8) or mifamurtide (the agonist of Nod2)) were incorporated and had their surface covered by an antigen (P24 from HIV). It was shown that the NPs were liberated from the patch and reached the nucleated epithelial cell (across the keratin layer, which exists in mice but not in humans). The formulation was moderately toxic in vitro for dendritic cells but not toxic at all for epithelial cells. The cytokine expression profile after the administration of the two formulations of NPs showed moderate expression of IFN-γ, IL-1, IL-6, IL-9, IL-13, and MIP-1 (CCL4), which demonstrates the efficient delivery of these adjuvants by the system used. This paper is interesting as it promotes a new way of administering antigens for adjuvanted vaccination. It has the benefit of promoting not only IgG production but also local IgA; this is not the case with vaccines administered by a systemic route.

## Figures and Tables

**Table 1:** Emerging viral zoonosis—years since the first described human infections.

Year	Virus (Human or Animal Disease)	Animal Reservoir	Epidemiology (Human)
1952 2007	*Chikungunya virus* (Chikungunya)	Primates	Endemic Epidemic (Southern Europe)
1952	*Zika virus* (Zika f.)	Primates	Endemic
1955	*Oropouche virus* (Oropouche f.)	Sloths	Epidemic
1967	*Marburg virus* (Marburg hemorrhagic f.)	Bats	Epidemic
2007 2023	*Monkeypox virus* (Mpox)	Rodents	Sporadic (Equatorial Africa) Endemic (worldwide)
1976	*Ebola virus* (Ebola hemorrhagic f.)	Rodents? Bats?	Epidemic
1983	HIV 1 and 2 (AIDS)	Primates	Endemic (worldwide)
1996	Prion (bovine spongiform encephalopathy)	Bovines	Disappeared
1997	Inluenzavirus A virus (Avian flu) several serovars: e.g., H5N1, H3N8	Birds (chickens)	Isolated cases
2003	SARS-Cov (SARS)	Civets and bats	Pandemic (disappeared)
2012	MERS-Cov (MERS)	Camels/bats?	Sporadic (Arabic peninsula)
2019	SARS-Cov 2 (COVID-19)	? (Bats?)	Endemic (worldwide)

f: fever; HIV: *Human Immunodeficiency virus*; AIDS: acquired immunodeficiency syndrom; SARS: severe acute respiratory syndrom; SARS-Cov: *Severe acute respiratory syndrome-related coronavirus*; COVID-19: coronavirus disease 2019; MERS-Coronavirus: *Middle-East-respiratory-syndrome-related coronavirus*; MERS: Middle East respiratory syndrome: ?: It is to to indicate that aimal origin still not identified.
